# The clinical translation of plasma p‐tau217 warrants higher urgency than additional efficacy validation studies

**DOI:** 10.1002/alz.70477

**Published:** 2025-08-05

**Authors:** Xiaolin Cui, Zhiming Lu

**Affiliations:** ^1^ Department of Clinical Laboratory Shandong Provincial Hospital Affiliated to Shandong First Medical University Jinan Shandong China

Dear Editor,

1

We read with interest the recent study by Wang et al. published in *Alzheimer's & Dementia*.^1^ Using the Lumipulse chemiluminescence assay, the authors demonstrated that plasma p‐tau217 and p‐tau217/Aβ42 ratios effectively identify abnormal Aβ and tau positron emission tomography (PET) status across clinical and community cohorts, highlighting their diagnostic value in early Alzheimer's disease (AD). The use of commercially available kits in this work provides critical insights for translating biomarker research into clinical practice, prompting essential considerations from a diagnostic laboratory perspective.

The global aging population has substantially expanded the at‐risk demographic for AD, necessitating scalable screening strategies. With approved monoclonal antibodies and preventive interventions now available, prioritizing clinical implementation over prolonged analytical validation could deliver earlier therapeutic benefits. Mounting evidence supports p‐tau217's diagnostic potential: Grande et al. reported that p‐tau217‐based biomarker panels effectively exclude imminent dementia in community settings,^2^ while Palmqvist et al. demonstrated plasma p‐tau217's superior 4‐year predictive accuracy compared to Aβ42/Aβ40 and neurofilament light chain (NFL).^3^ Comparative studies suggest plasma p‐tau217 performs equivalently to tau‐PET in predicting cognitive decline.^4^, ^5^ Regulatory approvals further underscore clinical viability, including United States Food and Drug Administration (FDA) Breakthrough Device Designations for Quanterix Simoa (March 2024), Roche Elecsys (April 2024), and Beckman Coulter's p‐Tau217/Aβ42 ratio (January 2025). However, excessive focus on analytical validation risks delaying patient access. We advocate immediate multicenter studies to establish standardized thresholds, followed by phased clinical implementation with real‐world monitoring.

Second, chemiluminescence should become the primary technical modality for AD marker detection and screening. Chemiluminescence immunoassays (CLIA) and single‐molecule array (Simoa) platforms currently represent the main technical approaches for plasma p‐tau217 clinical application, with head‐to‐head comparisons confirming their comparable performance.^4^ Universal access to disease screening mandates technical solutions emphasizing affordability, accessibility, and high‐throughput capacity. Notably, most AD screening candidates reside in primary care settings where CLIA instrumentation is widely available in district hospital laboratories at substantially lower costs than Simoa platforms. Furthermore, mature chemiluminescence systems offer full automation, high throughput, and operator familiarity, collectively positioning CLIA as the optimal approach for population‐level AD biomarker screening.

Third, implementing dual diagnostic thresholds may enhance early adoption of novel biomarkers. In the absence of validated standards and large‐scale cutoff determinations, novel blood markers require interpretive flexibility to mitigate misdiagnosis risks. Wang et al. achieved 92% specificity without sensitivity loss using dual cutoffs,^1^ while Brum et al.’s two‐step diagnostic strategy demonstrated that initial plasma p‐tau217 testing in mild cognitive impairment (MCI) patients reduces confirmatory testing requirements by 58%.^6^ We therefore propose incorporating “uncertainty intervals” during experimental clinical implementation to account for analytical variability and biological heterogeneity.

Critical challenges persist in plasma p‐tau217 research and clinical application. First, elevated p‐tau217 levels lack AD specificity. While p‐tau217‐based markers aid differentiation of AD from non‐AD cognitive deficits,^7^ their diagnostic accuracy for all‐cause dementia exceeds that for pure AD (81.0% vs. 76.8%).^2^ Elevated p‐Tau217 levels occur in human immunodeficiency virus (HIV) ‐associated neurocognitive disorders regardless of symptom status^8^ and may yield false positives in chronic kidney disease due to impaired protein clearance.^9^ These findings underscore the need for improved differential diagnostic strategies, potentially through multi‐marker panels.

Second, standardization of AD blood biomarkers demands urgent attention. Wang et al. observed inter‐cohort plasma biomarker variability that precluded unified threshold establishment.^1^ Baseline concentration discrepancies may arise from pre‐analytical factors (centrifugation speed, storage temperature), assay antibody batch variations, or matrix effects. This necessitates internationally recognized reference materials to ensure cross‐laboratory result comparability, alongside standardized protocols for sample processing and analytical procedures.

We commend these advancements and emphasize the imperative to translate validated biomarkers into clinical practice.

## CONFLICT OF INTEREST STATEMENT

The authors declare no competing interest. Author disclosures are available in the Supporting Information.

## Supporting information



Supporting Information
